# The global burden of cataracts and its attributable risk factors in 204 countries and territories: a systematic analysis of the global burden of disease study

**DOI:** 10.3389/fpubh.2024.1366677

**Published:** 2024-06-12

**Authors:** Dongyue Wang, Tong Tang, Peiheng Li, Jing Zhao, Bairong Shen, Ming Zhang

**Affiliations:** ^1^Department of Ophthalmology, West China Hospital, Sichuan University, Chengdu, China; ^2^Institutes for Systems Genetics, Frontiers Science Center for Disease-related Molecular Network, West China Hospital, Sichuan University, Chengdu, China; ^3^Center for High Altitude Medicine, West China Hospital, Sichuan University, Chengdu, Sichuan, China; ^4^Division of Thyroid Surgery, Department of General Surgery, West China Hospital, Sichuan University, Chengdu, China

**Keywords:** cataract, risk factors, global burden of disease study 2019, particulate matter pollution, smoking, high fasting plasma glucose, high body mass index

## Abstract

**Introduction:**

The global distribution and trends in the attributable burden of cataract risk have rarely been systematically explored. To guide the development of targeted and accurate cataract screening and treatment strategies, we analyzed the burden of cataract disease attributable to known risk factors.

**Method:**

This study utilized detailed cataract data from the Global Burden of Disease e 2019, and we analyzed disability-adjusted life years (DALYs) e each risk factor from 1990 to 2019. Additionally, we calculated estimated annual percentage changes (EAPCs) during the study period.

**Results:**

The results revealed that from 1990−2019, the global age-standardized DALYs of e attributable to particulate matter pollution, smoking, high fasting glucose plasma and high BMI showed steady downward trends (1990−2009: EAPC = −0.21 [−0.57 −0.14]); 2000−2009: EAPC = −0.95 [−1.01 −0.89]; 2010−2019: EAPC = −1.41 [−1.8 −1.02]). The age-standardized DALYs and mortality caused by each risk factor were highest in the low-middle sociodemographic index (SDI) region (EAPC = −1.77[(−2.19–−1.34)]). The overall disease burden of cataracts is lower in males than in females. When analyzing the EAPCs of cataract disease burden for each risk factor individually, we found that the age-standardized disability-adjusted life years caused by particulate matter pollution and smoking decreased (PMP1990-2009: EAPC = −0.53 [−0.9–−0.16]; 2000−2009: EAPC = −1.39 [−1.45--1.32]; 2010−2019: EAPC = −2.27 [−2.75–−1.79]; smoking 2000 to 2009: EAPC = −1.51 [−1.6–−1.43], 2009 to 2019: EAPC = −1.34 [−1.68–−1])), while high fasting plasma glucose and high body mass index increased annually (HFPG1990 to 1999: EAPC = 1.27 [0.89−1.65], 2000 to 2009: EAPC = 1.02 [0.82−1.22], 2010−2019: EAPC = 0.44 [0.19−0.68]; HBMI 1990 to 1999: EAPC = 1.65 [1.37−1.94], 2000 to 2009: EAPC = 1.56 [1.43−1.68], 2010−2019: EAPC = 1.47 [1.18−1.77]).

**Disscussion:**

The burden of cataracts caused by ambient particulate matter and smoking is increasing in low, low-middle SDI areas, and specific and effective measures are urgently needed. The results of this study suggest that reducing particulate matter pollution, quitting smoking, controlling blood glucose, and lowering BMI could play important roles in reducing the occurrence of cataracts, especially in older people.

## 1 Introduction

Cataract is defined as the loss of transparency of the lens, resulting in changes in refractive properties and increased light scattering, resulting in blurred vision or blindness (1). Cataracts are one of the leading causes of blindness worldwide (1, 2). The impact of cataracts on vision loss, particularly in older age groups, can significantly impact an individual’s quality of life (3) by exacerbating the possibility of dementia (4), falls (5) and road traffic accidents (6).

Although cataracts can be treated with simple and cost-effective surgery, one of the e challenges facing global ophthalmology today remains the high risk of operable cataract blindness, especially in developing countries (7, 8).

The 2017 Global Burden of Disease (GBD) study reported that cataracts are the second largest burden of eye disease (8 million) resulting in disability-adjusted life years (DALYs), just behind near vision loss (9.8million) (9).

Globally, the number of DALYs due to cataracts increased by 91.2% globally from 1990 to 2019. Previous study also approved that aging, female sex, and lower socioeconomic status were associated with a higher cataract burden (10). Previous studies have shown that areas with a low sociodemographic index (SDI) have a greater burden of cataracts (11).

However, detailed information about cataract disease burden by region, sex, and age group at the specific risk factor level remains elusive, hampering cataract prevention and control. GBD research data from 1990 to 2019 show that the prevalence of risk factors has changed significantly over the past 30 years. Summary exposure values (SEVs) for household air pollution from smoking and solid fuels decreased in all SDI quintiles. In contrast, the SEV of environmental particulate matter pollution increased significantly (10). Findings from a UK Biobank study revealed a correlation between higher ambient exposure to PM 2.5 and an increased likelihood of future cataract surgery (12). The number of deaths and DALYs caused by high body mass index (BMI) increased significantly globally. A pattern of a temporary increase in disease burden is associated with a high BMI in areas with the lowest SDI (13). Previous Mendelian randomization studies have associated genetically higher BMI and susceptibility to type 2 diabetes with an increased likelihood of age-related cataracts (14). High fasting plasma glucose (HFPG), also occurs in males and in areas with lower SDI, is an important factor in increasing global and regional disease burden (15).

Changes in these risk factors that contribute to cataract disease burden are primarily influenced by economic development and demographic changes. Therefore, in addition to delineating the overall pattern of cataract burden, timely studies are needed to comprehensively examine the impact of various risk factors on cataract burden. In this study, we analyzed the burden of cataracts attributable to four risk factors from 1990 to 2019 by SDI, age, and sex to reveal the different trends and distribution characteristics of the burden caused by each risk factor.

## 2 Materials and methods

### 2.1 Data sources and definitions

The GBD study established a freely accessible database containing data on estimated attributable burdens obtained through standardized methods for various risk factors in all countries. GBD 2019 includes more than 3.5 billion estimates for 369 diseases and injuries, 286 causes of death, and 87 behavioral, environmental, occupational, and metabolic risk factors in 204 countries and territories from 1990 to 2019.

The GBD 2019 estimates the global burden by age and sex of 369 diseases and injuries and 87 risk factors in 204 countries and territories between 1990 and 2019 by quantifying the health costs of premature death and nonfatal disability (16).

The sociodemographic index (SDI) was developed by GBD researchers and calculated as the geometric mean of these indices: total fertility rate in those under 25 years old, mean education for those aged 15 years or older, and lag-distributed income *per capita*. The global countries and territories were categorized into five super regions according to the quintiles of country-level estimates of SDI for the year 2019: low-SDI (0 ∼ <0.455), low-middle-SDI (0.455 ∼ <0.608), middle-SDI (0.608 ∼ <0.690), high-middle-SDI (0.690 ∼ <0.805), and high-SDI (0.805–1).^1^

The particulate matter pollution in GBD 2019 included both outdoor and indoor PM 2.5 pollution. PM 2.5 refers to particulate matter with an aerodynamic diameter ≤ 2.5 μm. GBD 2019 identified different sources of PM 2.5: outdoor PM 2.5 pollution, also known as ambient particulate matter pollution (APMP), due to exposure to PM 2.5 in outdoor air, and indoor PM 2.5 pollution, also known as solid particulate matter pollution (HAP) fuel, which refers to exposure to PM 2.5 due to the use of solid cooking fuels (wood, coal, charcoal, agricultural residues and manure). The theoretical minimum risk exposure levels for APMP and HAP are evenly distributed between 2.4 μg/m^3^ and 5.9 μg/m^3^ and represent the level that minimizes risk at the population level or captures the maximum attributable burden (17).

According to the GBD Project, a current smoker is an individual who currently uses any smoked tobacco product on a daily or occasional basis. Ex-smokers included individuals who had quit smoking for at least 6 months when possible or according to the definition used in the survey (18).

High fasting plasma glucose was defined as any level of FPG above the theoretical minimum-risk exposure level (TMREL), which is 4.8–5.4 mmol/L in the GBD study (16).

A high BMI is defined as a BMI greater than or equal to 25 kg/m2 for people over 20 years old, and a BMI of 20–25 kg/m2 is considered the theoretical minimum risk exposure level (19).

This study of cataract burden and its risk factors did not involve human subjects, and data were taken from the Global Health Data Exchange GBD Results Tool (See Footnote 1). This study complies with the Guidelines for Accurate and Transparent Reporting of Health Estimates (GATHER) guidelines for reporting health estimates (20). The detailed diagnostic and estimation methods for GBD 2019 have been published previously (16). In our study, we obtained publication estimates of DALYs with “cataracts” from the “all cause” category of the GBD website across 204 countries and territories. The statistical code used for GBD estimation is publicly available on the internet.

The GDB study was approved by the institutional review board of the University of Washington. Original data were collected with informed consent from the study participants or with a waiver from the institutional review board. As this was a secondary analysis of publicly available data, no further review by an institutional review board was required following the data use agreement of The Institute for Health Metrics and Evaluation.

### 2.2 Statistical analysis

The number of deaths or DALYs, age-standardized rates (ASRs) and estimated annual percentage change (EAPC) with a 95% uncertainty interval (UI) were adopted to quantify the cataract burden attributable to risk factors. The ASR, as a weighted mean of the age-specific rates, was considered necessary when comparing populations from different locations or for the same population over time in which the age profiles changed accordingly. The ASR was calculated as:


$$ASR=\frac{\sum_{i=1}^{A}a_{i}w_{i}}{\sum_{i=1}^{A}a_{i}}\times 100,000$$

$a_{i}$ : specific age ratio, $w_{i}$ : number of people (or weight).

of selected standard population, 100,000: per 100,000 population (21). The EAPC, which is widely accepted to reflect the annual change in rate over a specific period, was calculated based on the linear regression model y = α + βx + ε, where y = ln(ASR) and x = calendar year. Then, the EAPC can be obtained from 100 × (exp(β) − 1), as well as its 95% UI (22). If both the EAPC estimation and the lower limit of the 95% UI were positive, then the ASR showed a non-decreasing trend. Conversely, if both the EAPC estimation and the upper limit of the 95% UI were negative, the ASR exhibited a non-increasing trend. Under other conditions, the ASR was considered stable. A locally weighted regression analysis was applied to identify the association between the EAPC and the SDI. Z score hierarchical cluster analysis and Pearson’s test were performed to assess the patterns of ASR of risk factor-related DALYs in 204 countries and territories and 21 GBD regions and their temporal trends. Linear regression analysis is used to identify best-fit points and evaluate trends between these consecutive points.

Furthermore, we evaluated the relationship between the SDI and cataract disease burden. A two-sided *p* value of less than 0.05 was considered to indicate statistical significance. All the statistical analyses were performed using R version 4.3.1 (23).

## 3 Results

### 3.1 Overall impact of risk factors on cataract burden

**Figure 1:**
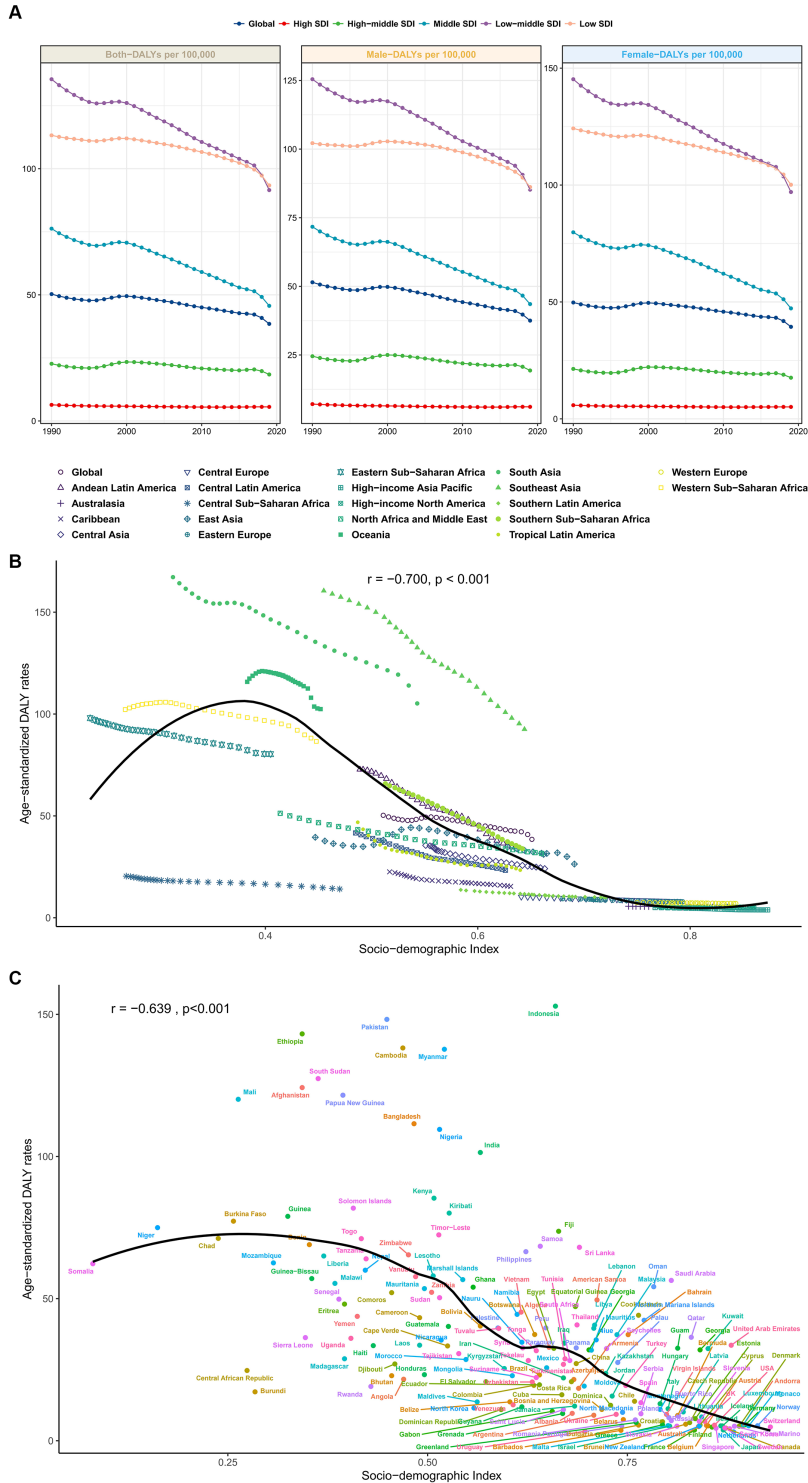
**(A)** From 1990 to 2019, the global age-standardized DALYs of cataracts attributable to risk factors showed steady downward trends in males, females and both sexes. **(B)** Changes in the SDI age-standardized DALY rates by region from 1990 to 2019. Areas above the solid black line have a greater burden than expected (based on the SDI), while those below the line have a lower burden than expected. **(C)** Association between age-standardized DALY rates and SDI across countries in 2019. DALYs, disability-adjusted life years; SDI, sociodemographic index.

From 1990 to 2019, the global age-standardized DALYs of cataracts attributable to risk factors showed steady decreasing trends (Figure 1A).

In 2019, among the five SDI quintiles, the age-standardized DALYs of cataracts due to risk factors were highest in low-middle-SDI areas (135.47 [82.97–196.56]), followed by low-SDI areas (113.23 [67.61–165.67]). High-SDI areas had the lowest disease burden (6.39 [4.08–9.52]; Supplementary Tables S1–S3). This trend was similar among males and females, as shown in Figure 1, with males having a lower overall disease burden of cataracts than females.

Figures 1B,C shows scatter plots of the SDI and DALYs among cataract patients in 22 countries/regions and 204 countries/regions. Age-standardized rates of cataract-related DALYs were lower in regions with SDIs larger than 0.6 (Figure 1B). The age-standardized rate of cataract-related DALYs was greater in low-SDI areas and decreased with increasing SDI. Figure 1C shows the association between age-standardized DALY rates and the SDI across countries in 2019. Across countries, age-standardized DALY rates increased with increasing SDI up to an SDI of approximately 0.38 but then declined with increasing SDI.

### 3.2 Cataract burden stratified by age and sex

**Figure 2:**
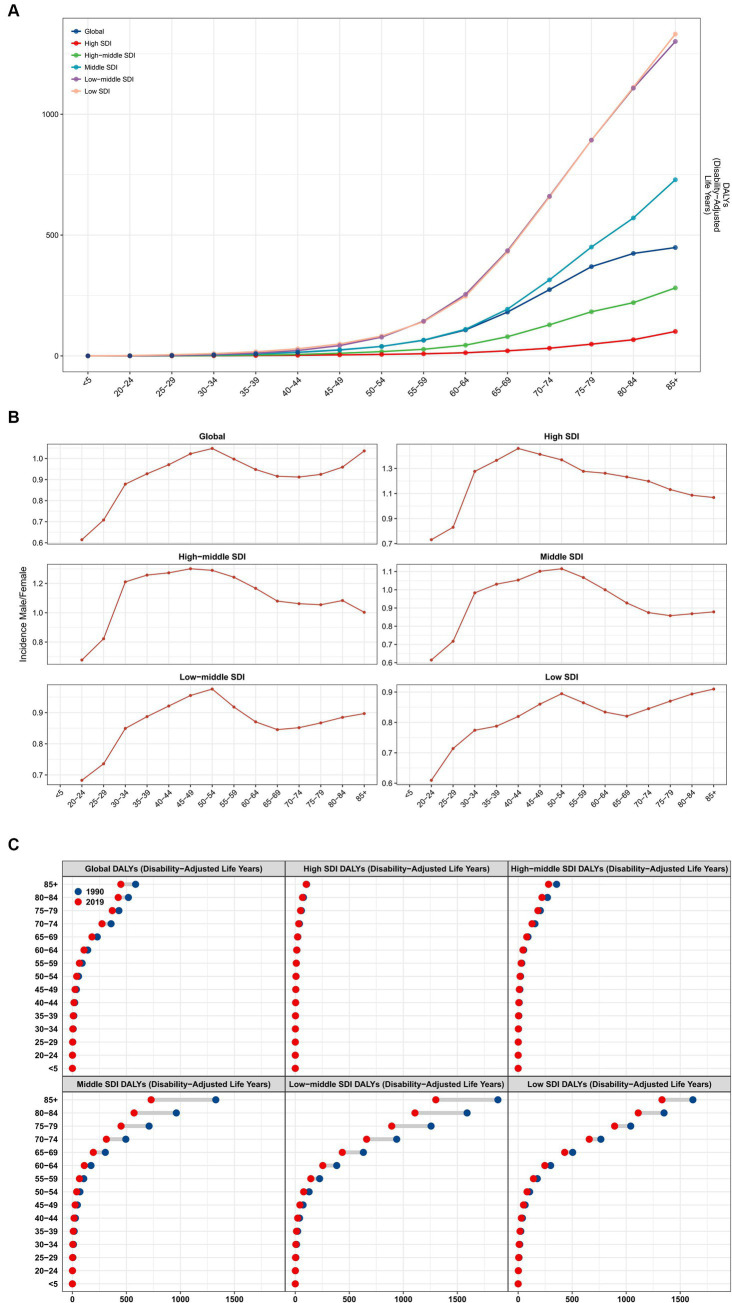
**(A)** Cataract DALY age distribution in SDI countries/regions, analysis of the **(B)** male/female ratio and **(C)** comparison between 1990 and 2019. EAPC, estimated annual percentage change; SDI, sociodemographic index.

From the perspective of age distribution, cataract DALYs increase with age, and the growth rate after the age of 60 years in low-middle- and low-SDI countries/regions seems to be higher than that in other SDI countries/regions (Figure 2A).

Subsequent analysis of the male/female ratio revealed that after the age of 20, the male/female ratio continued to approach 1, peaking at the age of 50–54 and then declining with age (Figure 2B).

By comparing the 1990 data with the 2019 data (Figure 2C), we found that overall, global cataract DALYs declined significantly among people over 60 years old, with almost no significant change in high-SDI countries/regions. As the SDI decreases, the changes gradually become apparent until middle-low-SDI countries. The change in DALYS of cataract burden in low-SDI countries from 1990 to 2019 was smaller than that in low-medium-SDI and medium-SDI countries/regions.

### 3.3 Contributions of risk factors to cataract burden by SDI, sex and age

From 1990 to 2019, the DALYs attributed to risk factors for cataracts showed a steady downward trend. In terms of overall risk factors, the age-standardized rate of cataract EAPC caused by risk factors decreased significantly from 2010 to 2019, and with the continuous development of society, the decrease in the EAPC continued to accelerate (1990–2009: EAPC = −0.21 [− 0.57–0.14]; 2000 to 2009: EAPC = −0.95 [−1.01–−0.89]; 2010–2019: EAPC = −1.41 [−1.8–−1.02]; Table 1).

**Table 1:** The temporal trends of cataract age-standardized DALYs attributed to risk factors across different SDI regions from 1990 to 2019.

EAPC	Years	All risk factors	High fasting plasma glucose	High body-mass index	Particulate matter pollution	Smoking
Global EAPC (95% CI)	1990–1999	−0.21 (−0.57–0.14)	1.27 (0.89–1.65)	1.65 (1.37–1.94)	−0.53 (−0.9--0.16)	0 (−0.3–0.3)
	2000–2009	−0.95 (−1.01--0.89)	1.02 (0.82–1.22)	1.56 (1.43–1.68)	−1.39 (−1.45--1.32)	−1.51 (−1.6--1.43)
	2010–2019	−1.41 (−1.8--1.02)	0.44 (0.19–0.68)	1.47 (1.18–1.77)	−2.27 (−2.75--1.79)	−1.34 (−1.68--1)
High SDI EAPC (95% CI)	1990–1999	−0.93 (−1.13--0.74)	−0.25 (−0.68–0.18)	0.93 (0.91–0.94)	−4.84 (−5.31--4.37)	−1.23 (−1.29--1.18)
	2000–2009	−0.6 (−0.62--0.58)	1.57 (1.49–1.65)	0.36 (0.32–0.39)	−6.09 (−6.18--6.01)	−1.52 (−1.57--1.48)
	2010–2019	0.22 (0.13–0.3)	1.28 (1.21–1.34)	0.99 (0.9–1.08)	−3.52 (−3.98--3.07)	−0.79 (−0.91--0.67)
High-middle SDI EAPC (95% CI)	1990–1999	0.19 (−0.57–0.95)	1.54 (0.96–2.13)	1.16 (0.85–1.48)	−0.47 (−1.47–0.55)	0.76 (0.24–1.29)
	2000–2009	−1.19 (−1.34--1.04)	1.52 (1.1–1.94)	0.87 (0.83–0.91)	−2.74 (−2.95--2.54)	−1.34 (−1.45--1.22)
	2010–2019	−0.85 (−1.35--0.34)	−0.14 (−0.53–0.25)	1.17 (0.88–1.46)	−2.68 (−3.44--1.92)	−0.17 (−0.63–0.29)
Middle SDI EAPC (95% CI)	1990–1999	−0.83 (−1.23--0.42)	0.93 (0.56–1.3)	1.65 (1.33–1.97)	−1.36 (−1.78--0.93)	0.12 (−0.26–0.5)
	2000–2009	−1.77 (−1.85--1.7)	0.25 (0.07–0.44)	1.33 (1.23–1.43)	−2.42 (−2.51--2.33)	−1.92 (−2.03--1.81)
	2010–2019	−2.48 (−2.89--2.07)	−0.37 (−0.63--0.11)	1.11 (0.79–1.43)	−3.86 (−4.39--3.33)	−1.89 (−2.23--1.54)
Low-middle SDI EAPC (95% CI)	1990–1999	−0.76 (−1.02--0.49)	0.68 (0.41–0.96)	1.89 (1.49–2.29)	−0.9 (−1.16--0.63)	−1.05 (−1.24--0.86)
	2000–2009	−1.3 (−1.34--1.25)	0.46 (0.28–0.63)	2.11 (1.75–2.48)	−1.5 (−1.54--1.46)	−2.19 (−2.26--2.11)
	2010–2019	−1.77 (−2.19--1.34)	0.39 (0.19–0.6)	1.75 (1.28–2.21)	−2.23 (−2.7--1.75)	−2.22 (−2.58--1.85)
Low SDI EAPC (95% CI)	1990–1999	−0.13 (−0.25--0.02)	1.07 (0.93–1.22)	1.43 (1.21–1.66)	−0.22 (−0.33--0.11)	−0.42 (−0.48--0.36)
	2000–2009	−0.47 (−0.5--0.44)	0.74 (0.59–0.88)	2.31 (2.15–2.47)	−0.59 (−0.62--0.56)	−0.98 (−1.01--0.95)
	2010–2019	−1.3 (−1.58--1.01)	0.13 (−0.01–0.26)	1.24 (1.11–1.38)	−1.49 (−1.8--1.17)	−2.24 (−2.49--1.99)

DALYs, disability-adjusted life years; EAPC, estimated annual percentage change; CI, certainty interval; SDI, Sociodemographic Index.

**Figure 3:**
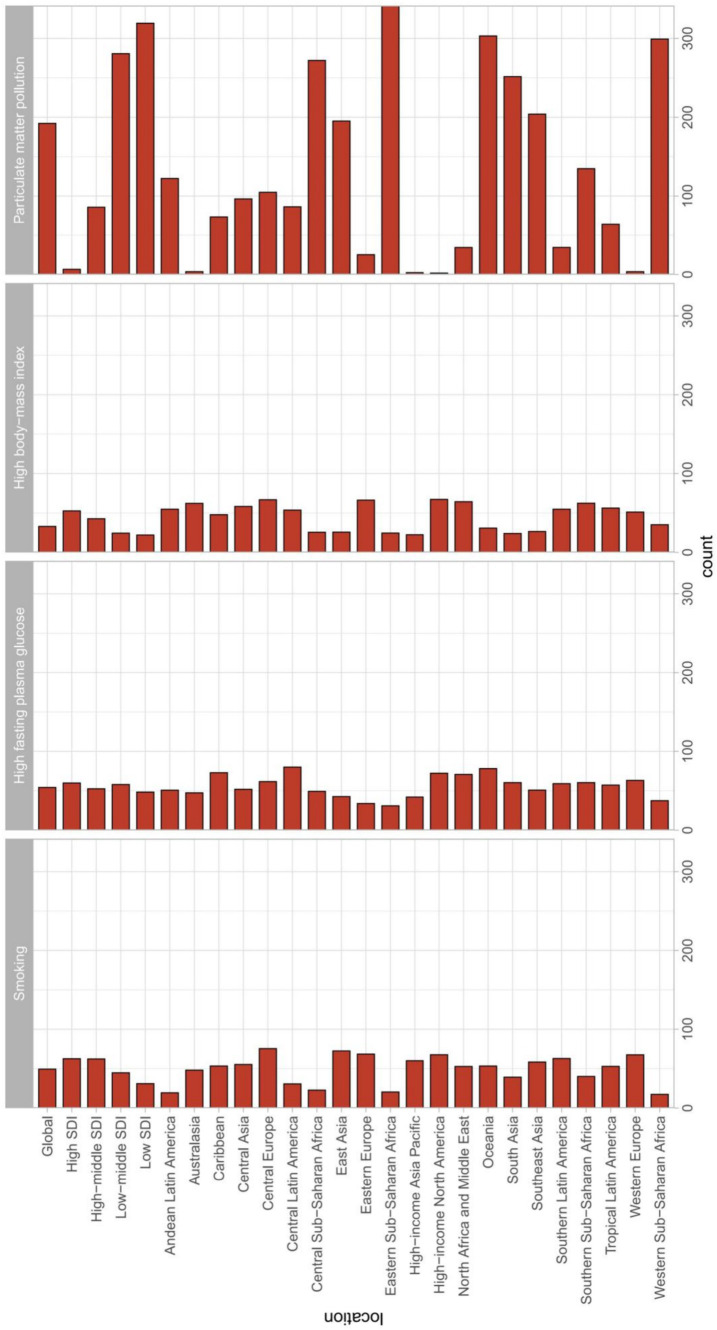
EAPC in DALYs attributable to risk factors for cataracts between 1990 and 2019 globally among different SDI quintiles and regions. EAPC, estimated annual percentage change; DALYs, disability-adjusted life years.

As shown in Figure 3, from a global perspective, the main risk factor leading to cataract disease burden in 2019 was particulate matter pollution, followed by smoking, high fasting plasma glucose (HFPG) and high body mass index (BMI).

**Figure 4:**
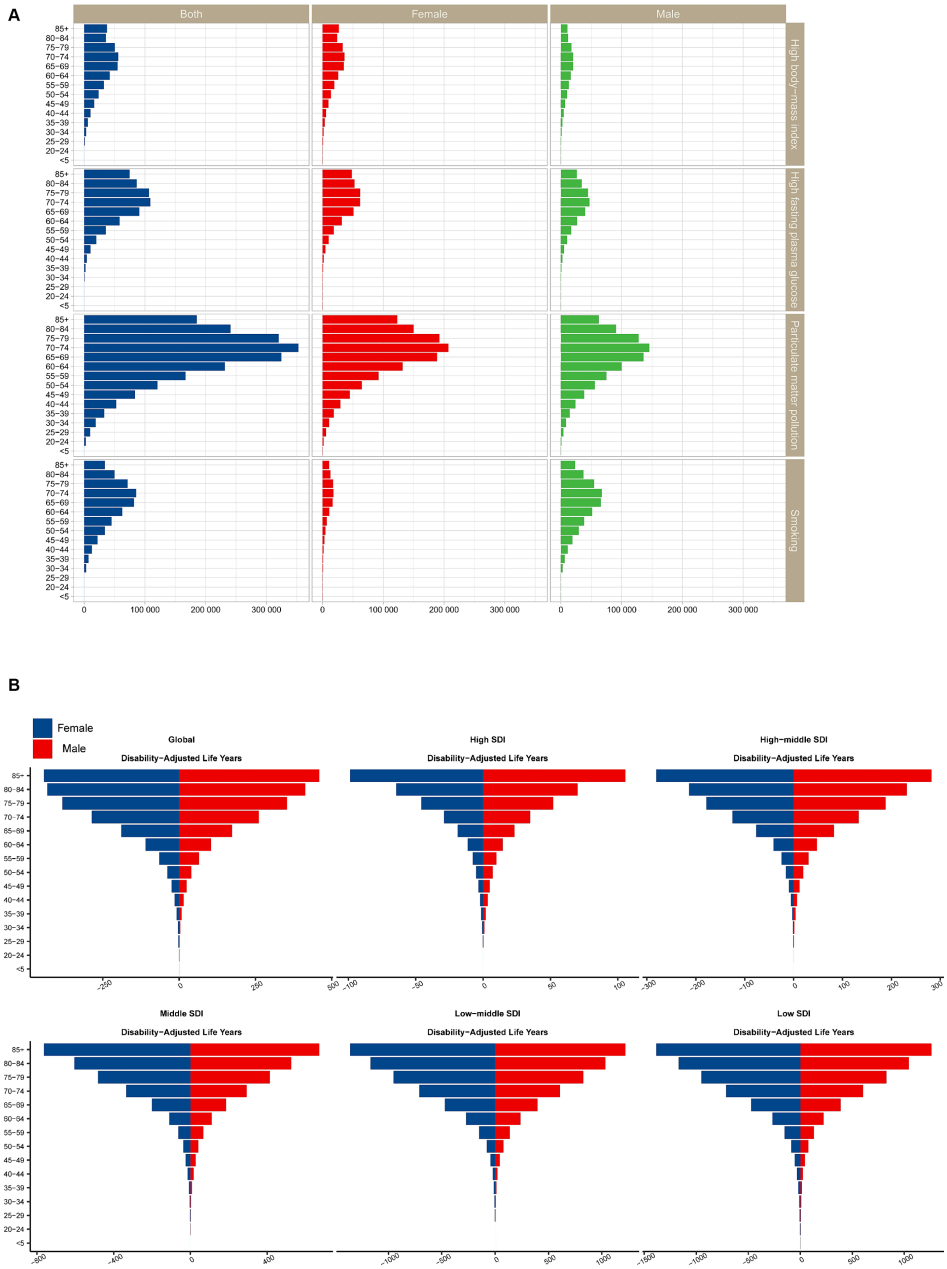
**(A)** Proportions of cataract risk across age groups in both sexes. **(B)** Trends in cataract risk across SDI countries/regions stratified by sex. SDI, sociodemographic index.

From the perspective of population age, as age increases, the burden of cataract disease caused by any type of risk factor increases (Figure 4A).

It is important to note that when analyzing the EAPC for cataract disease burden for each risk factor separately, we found a downward trend in age-standardized disability-adjusted life years due to particulate matter pollution and smoking, while high fasting plasma glucose and high body mass index increased over time.

### 3.4 Particulate matter pollution

Globally, particulate matter pollution is the leading risk factor responsible for the burden of cataracts (Figure 4B). Almost all SDI regions showed a downward trend in DALYs. The largest decreases in age-standardized disability-adjusted life years due to particulate matter pollution occurred in low-middle-SDI regions. From the perspective of regional classification, particulate matter pollution causes cataract disease burden in low-middle-SDI and low-SDI regions, which is much greater than that in other SDI regions. With the continuous development of society, the decrease in the EAPC continued to accelerate (1990–2009: EAPC = −0.53 [−0.9–−0.16]; 2000–2009: EAPC = −1.39 [−1.45–−1.32]; 2010–2019: EAPC = −2.27 [−2.75–−1.79]).

In terms of sex, females are more likely to suffer from cataracts due to particulate matter pollution than males are. In 2019, the age-standardized DALY for cataracts due to particulate matter pollution was slightly greater in females than in males.

Moreover, among all age groups (Figure 4A), particulate matter pollution had a greater impact on cataracts among DALYs. In particular, for the population over 60 years old, those aged 70–74 years had the highest DALY rate, while a lighter burden appeared for those under 50 years old.

### 3.5 Smoking

In terms of regional classification, the regions with the largest decreases in age-standardized disability-adjusted life years due to smoking were low-middle-SDI regions and middle-SDI regions (Figure 3). The decrease in the EAPC due to smoking in other regions slowed in the past decade (2000 to 2009: EAPC = −1.51 [−1.6–−1.43], 2009 to 2019: EAPC = −1.34 [−1.68–−1]; Table 1).

Additionally, the impact of smoking on cataracts was much greater in men than in women (Figure 4B). In 2019, the disease burden attributable to smoking was greater in men than in women across all age groups and SDI regions. The global cataract DALY rate due to smoking increased with age, peaked among those aged 70–74 years, and then decreased (Figure 4A).

### 3.6 High fasting plasma glucose

From the perspective of regional classification, HFPG leads to smaller differences in age-standardized disability-adjusted life years in different countries/regions (Figure 3). The greatest burden of cataract disease was in low-middle-SDI regions, followed by low-SDI regions. It should be noted that from 1990 to 2019, the age-standardized disability-adjusted life-year rate for cataracts caused by HFPG showed an increasing trend (1990 to 1999: EAPC = 1.27 [0.89–1.65], 2000 to 2009: EAPC = 1.02 [0.82–1.22], 2010–2019: EAPC = 0.44 [0.19–0.68]; Table 1).

### 3.7 High body mass index

From the perspective of regional classification, a high BMI leads to smaller differences in age-standardized disability-adjusted life years in different SDI countries/regions (Figure 3). From 1990 to 2019, the age-standardized disability-adjusted life-year rate of cataracts caused by high BMI also showed an upward trend (1990 to 1999: EAPC = 1.65 [1.37–1.94], 2000 to 2009: EAPC = 1.56 [1.43–1.68], 2010–2019: EAPC = 1.47 [1.18–1.77]; Table 1). In terms of sex, we found that a high BMI had a stronger impact on cataract burden in females than in males, and a high BMI caused a greater difference in cataract DALYs among females in different SDI areas than among males in different SDI areas (Figure 4B).

## 4 Discussion

In this study, we comprehensively analyzed the current burden, trends, and risk factors for cataract DALYs at the global and regional levels from 1990 to 2019 based on the GBD 2019 study. This study provides further evidence for the implementation of relevant policies and strategies to prevent and control the increase in cataract burden in the future by longitudinally and cross-sectionally comparing risk factor exposure levels and the disease burden that each risk factor can cause.

This study revealed that, in terms of overall risk factors, the age-standardized rate of cataracts caused by risk factors decreased from 2010 to 2019, and with the continuous development of society, the decline in the EAPC continued to accelerate. When analyzing the EAPC of cataract disease burden for each risk factor separately, we found that the age-standardized disability-adjusted life years caused by particulate matter pollution and smoking showed a downward trend, while high fasting plasma glucose and high body mass index increased annually.

The age-standardized incidence of cataracts is decreasing globally. However, the incidence of cataracts varies greatly in different SDI regions, and the burden of cataracts caused by major risk factors, especially particulate matter pollution and smoking, varies greatly among SDI regions. Research shows that in areas with higher SDI levels, the burden of disease caused by risk factors is relatively small. In contrast, the cataract burden caused by risk factors is relatively high in low-SDI areas. SDI levels are directly related to population health, and the large wealth gap in many parts of the world has implications for health equity.

Previous studies have shown that in areas with lower SDI and lower health literacy, inadequate medical resources and preventive measures may lead to a greater burden of cataract disease. Moreover, residents of more socioeconomic areas are more likely to have greater health literacy, better access to medical care, healthier diets and regular exercise, which may help reduce disease burden (24). A previous study suggested that lower income may be associated with an increased risk of a variety of vision-threatening diseases and may adversely affect ophthalmologists’ professional diagnostic and treatment approaches (25).

Interestingly, we found that the cataract disease burden was greater in low-middle-SDI regions (135.47 [82.97–196.56]) than in low-SDI regions (113.23 [67.61–165.67]). This may be due to socioeconomic development, as cataract risk factors such as high body mass index (BMI), unhealthy diet, low physical activity, environmental pollutants, and smoking have increased significantly, but the health system has failed to keep up with the relevant population health needs (26, 27).

In addition, another possible explanation is that there are fewer medical institutions and health resources in low-SDI areas, which may lead to the underreporting of cataract data. In terms of sex, this study revealed that in most regions and age groups, the disease burden of cataract patients was slightly greater for females than for males. One possible explanation is that women have a greater incidence of cataracts and a longer life expectancy (28, 29). Another explanation may relate to gender inequality, with evidence that women are disadvantaged in areas such as education, employment opportunities, income distribution and health care (28). Previous research revealed that although 60% of cataract patients are female, males are 1.39 times more likely to undergo cataract surgery than females are (30). Women possess less family support and less control over their finances than men, which may prevent them from undergoing cataract surgery. For children with bilateral cataracts, girls are also less likely to undergo surgery than boys in low-income countries (31). Therefore, more emphasis should be placed on eye care services for women, and eliminating gender inequality is an important component in combating the global burden of cataracts.

In terms of risk factors, this study revealed that particulate matter pollution causes a greater disease burden for cataracts globally than smoking does, as previously recognized (2010–2019: EAPC = −2.27[−2.75–−1.79] vs. −1.34[−1.68–−1]). This may be attributed to two assumptions: first, the global population-weighted PM 2.5 concentration continued to increase rapidly from 2010 to 2015, reaching 44.2 μg/m3 in 2015 (32); second, population growth and aging, which led to increased air pollution and increased disease burden (33).

For males, in addition to particulate matter pollution, smoking is a major risk factor for cataract burden. Approximately 25% of men and 5.4% of women globally are smokers, posing a significant obstacle to tobacco control (34). Smoking not only affects the health of smokers but also has a greater impact on the health of people in the surrounding areas. In 2019, secondhand smoke was the sixth leading risk factor for death from cataracts. In addition, tobacco has a substantial negative impact not only on cataracts but also on cardiovascular disease, lung tumors, and fertility (35, 36). Many countries have adopted various approaches to reduce tobacco consumption with positive results, but this study indicated that more practical efforts are still needed (37).

For the HFGP (2010–2019: EAPC = 0.44 [0.19–0.68]) and high BMI (2010–2019: EAPC = 1.47 [1.18–1.77]), the associated increase in DALYs cannot be entirely attributed to population growth and aging. Other factors also contribute to the exacerbation of risk factors for HFPG and high BMI, and the two are correlated. The prevalence of overweight or obesity, physical inactivity, and unhealthy diet has been reported to be associated with an increased burden of hyperglycemia in recent decades (38, 39).

As the world is facing a serious situation of preventing and treating blindness, the World Health Organization (WHO) launched the global action “VISION 2020” (40). Over the span of 1990 to 2010, the number of individuals afflicted with cataract-induced blindness decreased by 11.4%, and the corresponding blindness rate decreased from 38.6 to 33.4% (41, 42).

Taken together, these results suggest the need to strengthen early cataract screening in specific groups (women in medium-low and low-SDI countries/regions). Furthermore, efforts to reduce cataract risk must be combined with comprehensive control strategies, including efforts to support early diagnosis and effective treatment. The results of this study suggest that reducing particulate matter pollution, quitting smoking, controlling blood sugar, and lowering BMI play important roles in reducing the occurrence of cataracts, especially in the older population.

To the best of our knowledge, this is the first study to use the latest data from GBD 2019 to comprehensively assess the disease burden of cataracts by calendar year, age, sex, location, socioeconomic status, and risk factors, which will be useful to the public as well as health policy makers. However, some potential limitations of our study should not be overlooked. First, based on GBD 2019, few data on the prevalence, incidence, and subtypes of cataracts are available, which limits the analysis of the results to a certain extent. Second, predictions rely heavily on the quality of registration data based on the original population. The sparsity of cataract data, especially in low-SDI regions, may affect the accuracy of the estimates. However, the GBD 2019 study utilized a number of powerful statistical tools to reduce this effect. Third, as a population epidemiological study, we were unable to obtain individual-level data, which are inevitably affected by confounding factors when calculating correlation coefficients. However, our results provide clinical scientists and socioeconomists with updated big data and a more comprehensive analysis of the burden of cataracts.

## 5 Conclusion

Our study delineates the overall pattern of cataract burden and emphasizes the need to bolster prevention and management efforts focused on reducing particulate matter pollution, quitting smoking, controlling blood sugar, and lowering BMI, especially for older people. More targeted and effective global public health strategies should be developed and implemented to control cataracts and their associated risk factors.

## Data availability statement

The original contributions presented in the study are included in the article/Supplementary material, further inquiries can be directed to the corresponding authors.

## Ethics statement

As the data were freely available, no ethical approval or informed consent was obtained.

## Author contributions

DW: Conceptualization, Writing – original draft. TT: Methodology, Software, Writing – review & editing. PL: Data curation, Formal analysis, Investigation, Writing – original draft. JZ: Writing – review & editing. BS: Supervision, Writing – review & editing. MZ: Funding acquisition, Supervision, Writing – review & editing.

## Footnotes

^1^http://ghdx.healthdata.org/gbd-results-tool
