# Supplementary material for: The global burden of cataracts and its attributable risk factors in 204 countries and territories: a systematic analysis of the global burden of disease study
Source: Front Public Health. 2024 Jun 12;12:1366677. doi: 10.3389/fpubh.2024.1366677 (PMC11199524; doi:10.3389/fpubh.2024.1366677)
Supplement: Supplementary file 1 [file Table_1.DOCX]

STable 1。The temporal trends of cataract age-standardized DALYs attributed to risk factors across different SDI regions, 1990 to 2019

| Characteristics | Number of DALYs * 1000 | | Age-standardized DALY rates (per 100,000 population) | | EAPC of Age-standardized DALY rates |
| --- | --- | --- | --- | --- | --- |
| Years | 1990  (95% UI) | 2019  (95% UI) | 1990  (95% UI) | 2019  (95% UI) | 1990-2019 |
| Global | 1908.76  (1179.86-2782.82) | 50.29  (31.2-73.2) | 3114.74  (1913.31-4613.6) | 38.49  (23.55-56.96) | -0.68  (-0.8,-0.56) |
| High SDI | 66.62  (42.52-99.94) | 6.39  (4.08-9.52) | 108.45  (64.6-169.89) | 5.58  (3.37-8.7) | -0.44  (-0.51,-0.37) |
| High-middle SDI | 225.78  (142.75-330.27) | 22.68  (14.31-33.27) | 372.85  (231.46-550.91) | 18.43  (11.43-27.25) | -0.42  (-0.61,-0.24) |
| Middle SDI | 676.73  (408.49-988.11) | 76.25  (45.68-111.36) | 1052.1  (636.02-1586.47) | 45.64  (27.36-68.65) | -1.49  (-1.65,-1.33) |
| Low-middle SDI | 706.44  (434.43-1033.42) | 135.47  (82.97-196.56) | 1150.41  (705.58-1694.11) | 91.53  (55.93-133.87) | -1.1  (-1.2,-1) |
| Low SDI | 232.22  (140.46-341.52) | 113.23  (67.61-165.67) | 429.42  (259.56-632.43) | 93.39  (57.03-136.44) | -0.49  (-0.58,-0.39) |
| Andean Latin America | 13.52  (7.86-20.43) | 72.8  (42.16-110.91) | 18.75  (10.77-28.79) | 34.65  (19.84-53.04) | -2.59  (-2.74,-2.43) |
| Australasia | 1.29  (0.84-1.92) | 5.65  (3.66-8.43) | 2.53  (1.5-3.93) | 5.02  (3-7.71) | -0.32  (-0.38,-0.26) |
| Caribbean | 5.64  (3.4-8.45) | 22.54  (13.57-33.79) | 7.94  (4.8-12.06) | 15.41  (9.32-23.34) | -1.22  (-1.3,-1.13) |
| Central Asia | 15.18  (9.12-22.85) | 35.53  (21.22-52.89) | 15.18  (9.25-22.84) | 24.12  (14.39-36.66) | -1.43  (-1.48,-1.37) |
| Central Europe | 14.17  (8.8-20.68) | 10.27  (6.42-14.9) | 17.67  (11.07-26.34) | 8.11  (5.09-12) | -0.83  (-0.86,-0.81) |
| Central Latin America | 30.9  (18.68-45.83) | 41.63  (25.15-61.55) | 52.75  (30.34-81.81) | 23.3  (13.4-36.02) | -1.92  (-2.01,-1.84) |
| Central Sub-Saharan Africa | 3.66  (2.16-5.51) | 20.44  (11.94-30.29) | 6.08  (3.51-9.05) | 14.11  (8.16-21.14) | -1.12  (-1.21,-1.03) |
| East Asia | 282.13  (174.28-411.31) | 39.5  (24.07-57.54) | 511.51  (306.19-766.38) | 26.36 (15.62-39.6) | -0.91  (-1.33,-0.49) |
| Eastern Europe | 24.43  (15.11-37.12) | 9.33  (5.83-14.17) | 27.17  (16.88-41.1) | 7.86  (4.9-11.96) | -0.65  (-0.69,-0.61) |
| Eastern Sub-Saharan Africa | 68.47  (40.73-101.87) | 97.99  (57.97-144.75) | 121.36  (72.79-178.85) | 80.32  (47.71-117.6) | -0.66  (-0.7,-0.62) |
| High-income Asia Pacific | 9.77  (6.29-14.85) | 5.03  (3.22-7.6) | 18.06  (10.31-29.32) | 3.87  (2.27-6.15) | -1  (-1.08,-0.92) |
| High-income North America | 19.27  (11.87-29.47) | 5.38  (3.33-8.19) | 34.46  (20.26-53.7) | 5.36  (3.19-8.33) | 0.15  (0.07-0.24) |
| North Africa and Middle East | 76.21  (46.86-115.64) | 51.3  (31.78-77.44) | 121.69  (74.39-187.25) | 31.6  (19.11-49.28) | -1.62  (-1.69,-1.55) |
| Oceania | 2.95  (1.84-4.27) | 115.86  (72.64-166.5) | 6.24  (3.93-9.19) | 102.44  (64.21-147.85) | -0.41  (-0.54,-0.28) |
| South Asia | 795.11  (487.22-1169.33) | 167.2  (101.44-244.25) | 1348.32  (822.4-2000.28) | 105.2  (64.1-155.58) | -1.29  (-1.41,-1.16) |
| Southeast Asia | 363.2  (218.75-539.26) | 160.5  (96.74-235.06) | 510.98  (303.68-754.42) | 92.52  (55.04-137.47) | -1.84  (-1.93,-1.75) |
| Southern Latin America | 6.04  (3.7-9.09) | 13.71  (8.41-20.6) | 8.42  (5.17-12.9) | 10  (6.16-15.3) | -1.05  (-1.1,-0.99) |
| Southern Sub-Saharan Africa | 17.01  (10.25-25.29) | 65.81  (39.9-98.48) | 17.39  (10.86-25.97) | 33.9  (21.19-50.72) | -2.34  (-2.45,-2.24) |
| Tropical Latin America | 37.05  (23.23-54.48) | 46.88  (29.14-69.23) | 54.37  (32.46-82.82) | 23.4  (13.9-35.8) | -1.9  (-2.13,-1.67) |
| Western Europe | 42.97  (26.98-65.23) | 7.48  (4.74-11.27) | 66.91  (38.68-106.14) | 7.02  (4.2-11.09) | -0.19  (-0.22,-0.17) |
| Western Sub-Saharan Africa | 79.79  (47.41-118.82) | 102.2  (61.01-151.46) | 146.95  (88.94-216.9) | 86.57  (52.07-128.06) | -0.57  (-0.68,-0.45) |
